# Three-Dimensional (3D) Conductive Network of CNT-Modified Short Jute Fiber-Reinforced Natural Rubber: Hierarchical CNT-Enabled Thermoelectric and Electrically Conductive Composite Interfaces

**DOI:** 10.3390/ma13112668

**Published:** 2020-06-11

**Authors:** Lazaros Tzounis, Markos Petousis, Marco Liebscher, Sotirios Grammatikos, Nectarios Vidakis

**Affiliations:** 1 Department of Materials Science and Engineering, University of Ioannina, 45110 Ioannina, Greece; 2 Mechanical Engineering Department, Hellenic Mediterranean University, Estavromenos, 71004 Heraklion, Greece; markospetousis@hmu.gr (M.P.); vidakis@hmu.gr (N.V.); 3 Institute of Construction Materials, Technische Universität Dresden, DE-01062 Dresden, Germany; 4 Group of Sustainable Composites, Department of Manufacturing and Civil Engineering, Norwegian University of Science and Technology, 2815 Gjøvik, Norway; sotirios.grammatikos@ntnu.no

**Keywords:** three-dimensional (3D) conductive network, conductive polymer composites (CPCs), thermoelectric elastomers, thermoelectric composites, hierarchical reinforcements, nanostructured interfaces

## Abstract

Jute fibers (JFs) coated with multiwall carbon nanotubes (MWCNTs) have been introduced in a natural rubber (NR) matrix creating a three-dimensional (3D) electrically conductive percolated network. The JF-CNT endowed electrical conductivity and thermoelectric properties to the final composites. CNT networks fully covered the fiber surfaces as shown by the corresponding scanning electron microscopy (SEM) analysis. NR/JF-CNT composites, at 10, 20 and 30 phr (parts per hundred gram of rubber) have been manufactured using a two-roll mixing process. The highest value of electrical conductivity (*σ*) was 81 S/m for the 30 phr composite. Thermoelectric measurements revealed slight differences in the Seebeck coefficient (*S*), while the highest power factor (*PF*) was 1.80 × 10^−2^ μW/m K^−2^ for the 30 phr loading. The micromechanical properties and electrical response of the composite’s conductive interface have been studied in peak force tapping quantitative nanomechanical (PFT QNM) and conductive atomic force microscopy (c-AFM) mode. The JF-CNT create an electrically percolated network at all fiber loadings endowing electrical and thermoelectric properties to the NR matrix, considered thus as promising thermoelectric stretchable materials.

## 1. Introduction

Carbon nanotubes (CNTs) have been widely used for the last three decades in various applications ranging from electronics, structural materials and composites, energy-harvesting devices, nanotechnology applications, etc. due to their unique electrical, mechanical and thermal properties [1,2,3]. Specifically, there have been numerous studies demonstrating that CNTs are an ideal carbon nanoallotrope material for fabricating multi-functional and smart nanocomposites. For instance, Tzounis et al. reported on the anisotropic electrical properties of polymer/CNT nanocomposites utilising a shear field for the CNT orientation [4]. Nanomodification of a polymer matrix could be achieved via conventional methods as for instance by dispersing conductive particles i.e., CNTs within an insulating matrix. However, it is worth mentioning that nanomodification and especially incorporation of nanofillers with different geometries e.g., spherical, tube-like, plate-like, etc. in a polymer matrix face some inherent process-related problems that could hinder the practical application of nanoparticles. For instance, in order to obtain an adequate electrical conductivity, the dispersion process has to be perfectly tuned to reach an optimal macro and nano-dispersion [5]. However, the viscosity increases abruptly with the increased weight fraction of CNTs in a polymer melt (i.e., melt-mixing or two-roll compounding, etc.) or in a polymer solution (i.e., solvent-mixing process). These phenomena complicate further the nanocomposite manufacturing.

The deposition of CNTs directly onto the surface of reinforcing fibers could be considered as a promising alternative approach to incorporate the nanofiller in the polymer matrix overcoming the aforementioned issues. Recently, the deposition of CNTs onto the surface of various fibrous materials has attracted the scientific interest of different groups working in the area of advanced fiber-reinforced polymer (FRP) composites [6]. In this manner, CNTs are introduced in the polymer matrix, while alleviating the critical problems encountered during the nanocomposite’s fabrication related to the high viscosity of the polymer melts, CNT agglomeration due to strong van der Waals forces, etc. [7]. Namely, some of the methods that have been used to create the hierarchical reinforcements include: i) conventional dip-coating [8], ii) electrophoretic deposition [9], iii) blade-coating [10] and iv) sizing mixtures containing CNTs directly applied to the fibers during the spinning process; all of them belonging to wet-chemical deposition methods, as well as high temperature CNT growth and deposition methods i.e., v) chemical vapor deposition (CVD) [11]. Till now, carbon [12], ceramic [13], glass [14] and natural fibres [15] have been employed as the reinforcement material for depositing CNTs following different techniques. The CNT-coated hierarchical fibers have been found to significantly improve the composite interfacial adhesion strength studied by single fiber pull-out and single fiber fragmentation micromechanical tests [7,16]. Moreover, fiber-reinforced polymer (FRP) composite laminates have been manufactured using CNT-coated fabrics showing an increased interlaminar shear strength (ILSS) properties [17]. Apart from the mechanical properties that have been found to be significantly increased, multi-functional properties are endowed to the final composites, due to the nanostructured CNT-rich interfacial regions. To that end, different functionalities e.g., temperature sensing [18], ultraviolet (UV)-light and resin cure-sensing [19], strain sensing for potential structural health monitoring (SHM) purposes [10], and thermoelectric energy harvesting [19] have been reported.

Natural FRP composites have been reported for the first time in 1908, where cellulose fibers were utillised as reinforcements for phenolic resins [20]. Natural fibers such as jute, flax or hemp have recently attracted considerable attention for manufacturing bio-friendly eco-composites, due to their unique intrinsic characteristics e.g., high specific mechanical properties at a very low cost, low density, and potential to be recycled [21]. As such, it has been realised that natural fibers could replace in many applications the synthetic ones, i.e., glass, carbon or aramid, due to their recyclable, biodegradable and non-polluting character contributing overall to the reduction of the carbon footprint and CO_2_ emissions. However, the use of natural fibers as polymer matrix reinforcements has to overcome some inherent drawbacks i.e., the poor compatibility and interfacial strength with the hydrophobic polymer matrices, as well as the relatively poor thermal stability [22]. In order to overcome these issues, a lot of studies exist with specific natural fiber surface modifications in order to improve the fiber/matrix adhesion strength [23].

The mechanical reinforcement of rubbers and other elastomer materials has been reported several times utilising fibrous fillers. However, a strong interaction between the rubber and the filler material should be achieved in order to realise high mechanical performance elastomer materials that can be manufactured into useful elastomer products [24]. Specifically, natural fibers e.g., pineapple leaf fiber [25], bamboo [26], short jute fiber [27], sisal/oil palm [28] and short coir fiber [29] have been introduced in elastomer compounds. Natural rubber/jute fiber composites have been reported by Tzounis et. al. where jute fibers (JFs) have been modified with a CNT thin layer as a macromolecular compatibilliser to achieve high mechanical performance hierarchical natural rubber (NR) composites [15].

In several cases, elastomers are exposed to environments where there is a temperature gradient. As such, their potential to harvest thermal energy and convert to electricity, otherwise known as thermoelectric or Seebeck effect is a very challenging field of research [30]. Thermoelectric materials are one of the potential candidates for thermal energy harvesting due to their ability to generate voltage upon being exposed to an ambient temperature difference. This is otherwise known as the Seebeck effect, which is the direct solid state conversion of thermal energy to electricity. An important physical property for thermoelectric materials is the Seebeck coefficient, which is defined as:

(1) $$S=\frac{\Delta V}{\Delta T}$$

where Δ*V* is the electric potential difference or thermoelectric voltage created by the temperature gradient, Δ*T*. The Seebeck coefficient is utillised further to derive the power factor [*PF* = *σ* × *S^2^* = *σ* × (Δ*V*/Δ*T*)*^2^*, *σ* is the electric conductivity], reported in several cases to compare the efficiency of different thermoelectric materials. Specifically, the Seebeck coefficient, *S*, is positive for p-type semi-conductors, and negative for n-type ones [31], while it is an intrinsic material property related to the material’s electronic properties [32]. Traditional thermoelectrics consist of low band gap semiconductors i.e., Bi_2_Te_3_, PbTe, etc., however there are issues related to toxicity, often consisting of rare elements and high cost for large scale thermoelectric generator (TEG) device fabrication [33].

Organic based thermoelectrics and more specifically CNT-based polymer nanocomposites have been reported already, while it is interesting to mention that TEG devices have been also demonstrated using serially interconnected polymer nanocomposite thermoelements [34]. Polymer/ SWCNT nanocomposites with high loadings (~50 wt.%) have reached very promising power factors (*σ* × *S^2^*) reaching values in the range of ~140 μW m^−1^ K^−2^ [35]. Till now, mainly solution and melt-processed CNT filled polymer thermoelectric nanocomposites have been reported [36], as well as short carbon fiber/polycarbonate composites [37]. Tzounis et. al. has reported the use of glass fiber/CNT hierarchical continuous reinforcements as thermoelectric reinforcements in model epoxy composites [19]. To the best of the author’s knowledge, the electrical and thermoelectric properties of NR/JF-CNT hierarchical composites have not been established yet.

Herein, we report for the first time the utilisation of hierarchical JF-CNT reinforcements to introduce electrical conductivity and thermoelectric properties to the respective NR bulk compounds. The 10 phr (parts per hundred gram of rubber) NR/JF-CNT composites have been shown already to be above the electrical percolation threshold, while by increasing the amount of JF-CNT to 30 phr brings the highest electrical conductivity (81 S/m). The electrical conductivity and thermoelectric behaviour endowed to the inherently insulating NR matrix is attributed to the JF-CNT with the CNT-CNT junctions overcoming the barrier of the insulating NR macromolecular chains.

## 2. Materials and Methods

A standard Malaysian Rubber (SMR-10) as the natural rubber matrix has been used in our study. Jute fiber continuous yarn as a bobbin was obtained from Gloster Jute Mills, (TD 4 grade, Howrah, India). Stearic acid was purchased from Acros Organics (Geel, Belgium, 97% purity). N-cyclohexyl-2-mercapto benzothiazole sulfonamide was received from Rhein Chemie (Mannheim Germany). Sulfur, zinc oxide, ethanol, toluene and sodium hydroxide were supplied by Sigma-Aldrich (Steinheim, Germany). MWCNTs of the NC 7000 that are commercially available were provided by Nanocyl company (Nanocyl S.A., Sambreville, Belgium). The MWCNTs according to the supplier specifications exhibit carbon purity >90%, average length 1.5 μm and diameters in the range of 10 nm. All the chemicals used in this study were analytical grade and employed in the different processes without further purification

The steps followed to create short JFs as well as to clean/activate the fiber surfaces and deposit the MWCNT thin layer have been in detail described in our previous study [15]. NR/JF-CNT composites were fabricated by initially preparing the NR mixture containing all the components for the cross-linking process. Briefly, the NR has been masticated in an open two-roll mixing mill (Polymix 110L, Servitech GmbH, Wustermark, Germany) at 70 °C for 5 min. The mastication process was carried out in order to reduce the NR viscosity and allow the processing/mixing of the components for the cross-linking as well as the short JF filler dispersion. Then, the appropriate amount of ZnO and stearic acid (shown in Table 1) were sequentially incorporated, while different amounts JF-CNT were added in the elastomer mixture followed by adding finally the accelerator and sulphur. The total two-roll mixing/compounding cycle occurred at 70 °C for a total duration of 15 min. The 10, 20 and 30 phr composites are denoted hereafter as NR/JF-CNT(10), NR/JF-CNT(20) and NR/JF-CNT(30), respectively. The mixture formulation is given in Table 1.

The stocks were cured finally at 150 °C under 4.9 MPa pressure using an optimum cure time. The optimum cure otherwise defined as vulcanization time was determined from curing studies that were performed using a rubber processing analyser (Scarabaeus SIS-V50, Langgöns, Germany) in the isothermal time sweep mode for all the samples at 150 °C for 60 min. The maximum rheometric torque (S_max_), scorch time (t_2_) and optimal curing time (t_90_) obtained from this study are recorded in Table 2. It has been found from the values presented in this table the maximum rheometric torque for the composites is always higher as compared with neat NR gum and the value is increasing with the increased JF-CNT loading.

**Figure 1:**
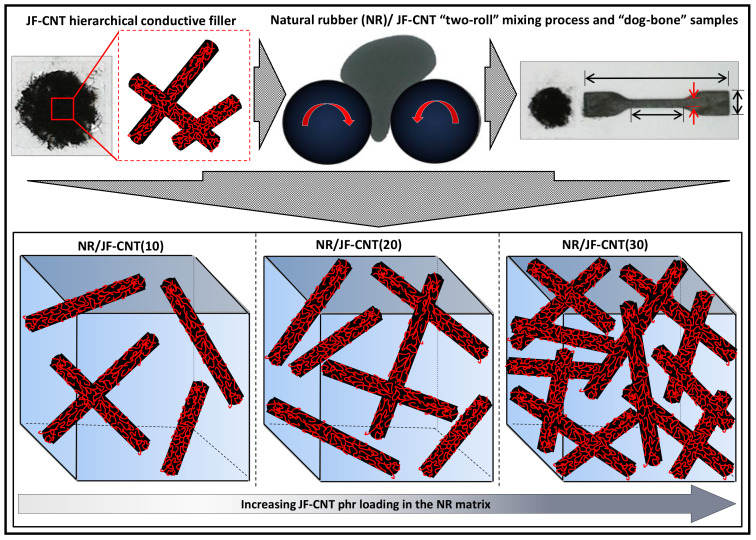
Schematic illustration of the two-roll mixing process for the fabrication of NR/JF-CNT composites and the resulting composites at different phr loadings. The final dog-bone shaped samples used for the tensile test experiments (dimensions of the samples according to the ASTM D412 (D 412) Tensile Strength Properties of Rubber and Elastomers, as well as the electrical conductivity and thermoelectric measurements are also depicted.

Figure 1 shows schematically the two-roll mixing process of the NR mixture with the JF-CNT and the resulting composite dog-bone shaped samples used for the tensile test investigations as well as the electrical and thermoelectric measurements. In the lower case of the figure, the final composites with the increase in the JF-CNT content randomly dispersed in the NR matrix is depicted.

A NEON 40 (Carl Zeiss AG, Oberkochen, Germany) field-emission scanning electron microscope (FE-SEM) operating at an accelerating voltage of 1.0 kV was used to study the fiber surface morphologies. Prior to the SEM analysis, the samples were sputter coated with a thin layer of platinum (~3 nm) in order to avoid charging effects.

In an attempt to visualize the electrically conductive interphases, single JF-CNT fibers were introduced in NR and ultrasmooth block surfaces were prepared perpendicular to the direction axis of the embedded fiber length using an ultramicrotome (Leica UC7, Leica Microsystems GmbH, Wetzlar, Germany) and a diamond knife for polishing (model cryotrim 45°) under cryo conditions (−120 °C). Afterwards, peak force tapping quantitative nanomechanical (PFT QNM) mode and current map were acquired by the PeakForce TUNA module with a Bruker ICON scanning probe microscope (Bruker Corporation, Santa Barbara, CA USA). Electrically conductive tips PPP-EFM supplied by NanoAndMore GmbH (Wetzlar, Germany) were employed and as measurement parameters, a scan rate of 0.5 Hz and a direct Voltage bias of 5 V were used. During the AFM measurements, the interface cross section was oscillated in the z-direction at 1 kHz moving the sample in the x–y direction simultaneously at a rate of 0.5 Hz. The PFT QNM mode adopts the Derjaguin–Muller–Toporov (DMT) model using 30% to 90% from the minimum force to the maximum peak force of every retraction curve for real-time curve fitting in order to acquire and map the DMT modulus over the scanning region of interest. In the current map images obtained, only the CNTs were visible which form the conductive paths at the fiber-matrix interfacial region. For the DMT modulus mapping, the accuracy and the level of the modulus measurement over the scanned area is guaranteed by controlling the sample deformation within the elastic deformation range. The current map and DMT modulus mapping were evaluated at 40 μm × 40 μm with 256 × 256 pixels at room temperature. The Nanoscope analysis software (Bruker, ver. 1.40) was used for the image analysis.

The electrical conductivity of NR/JF-CNT composites at 10, 20 and 30 phr loading in the form of rectangular-shaped samples was determined by measuring the sheet resistance (*Rs*) at room temperature using a four-point probe system (Ossila Ltd., Sheffield, UK). The *Rs* values were derived further to resistivity (*ρ*) and conductivity (*σ*) values via the well-known formula of *Rs* = *ρ*/*t* (*ρ*: resistivity equal to *σ* = 1/*ρ*, *t*: film thickness), considering the sample dimensions. It should be mentioned that the employed four-point probe system with four equally spaced, co-linear probes to the material (interelectrode probe distance/spacing: 1mm) is capable of delivering currents between 10 nA and 150 mA, and can measure voltages from as low as 100 μV up to 10 V, which results in a sheet resistance measurement range of 3 mΩ/square to 10 MΩ/square. The values reported are mean values of at least four measurements performed on each sample.

For the determination of the Seebeck coefficient, a self-made custom set-up was used. Details for the measurement could be found elsewhere [38]. In brief, NR/JF-CNT samples were mounted on two metal blocks, which enabled the generation of a temperature difference. For all measurements, one block was kept at room temperature (~25 °C), while the other one was heated at 125 °C allowing the generation of a temperature gradient. The generated thermovoltage (Δ*V*) was measured by a digital multimeter-voltmeter (Agilent 34401A6½, Agilent, Santa Clara, CA, USA), while the temperature of the two blocks was monitored with K-type thermocouples to determine precisely the temperature difference (Δ*T*). The Seebeck coefficient (*S*) was derived then from the ratio Δ*V*/Δ*T*.

## 3. Results and Discussion

### 3.1. Scanning Electron Microscopy Analysis of the Pristine Jute Fiber (JF) and JF-Carbon Nanotubes (CNT)

**Figure 2:**
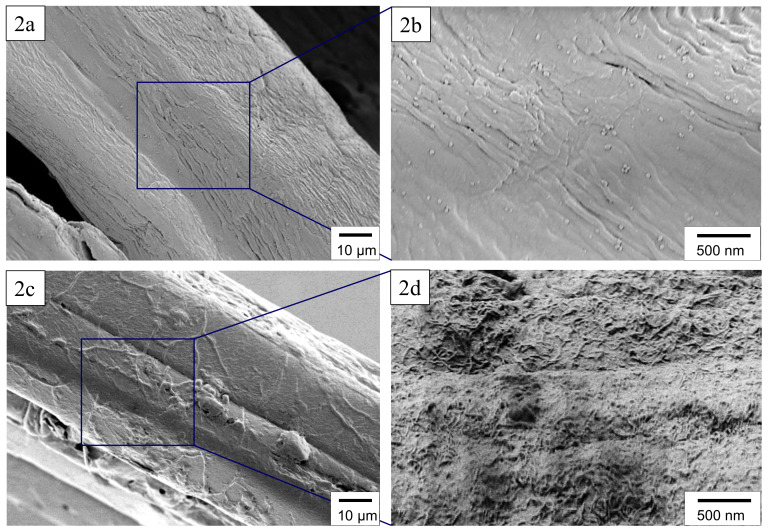
SEM images showing the surface morphology of pristine (**a**,**b**) and JF-CNT (**c**,**d**).

Figure 2 represents the SEM images of pristine JFs (a,b) and JF-CNT (c,d), respectively. The pristine JFs have undergone an alkali treatment removing inherent hemicellulosic impurities and/or other impurities like waxes, fats, lignin, pectin, all of which are part of the cementing which exists on the JF-surface in the as grown state. Therefore, the fiber morphology is very smooth with visible the natural fiber’s veins. JF-CNT hierarchical structure appears with a dense CNT-network consisting of interconnected CNTs covering fully the JF surface. The CNT deposited layer is considered to be the responsible conductive agent for the induced electrical conductivity and thermoelectric properties of the resulting NR-JF-CNT hierarchical composites.

### 3.2. Peak Force Tapping Quantitative Nanomechanical (PFT-QNM) and Conductive Atomic Force Microscopy (c-AFM) for the Interphase Investigation

**Figure 3:**
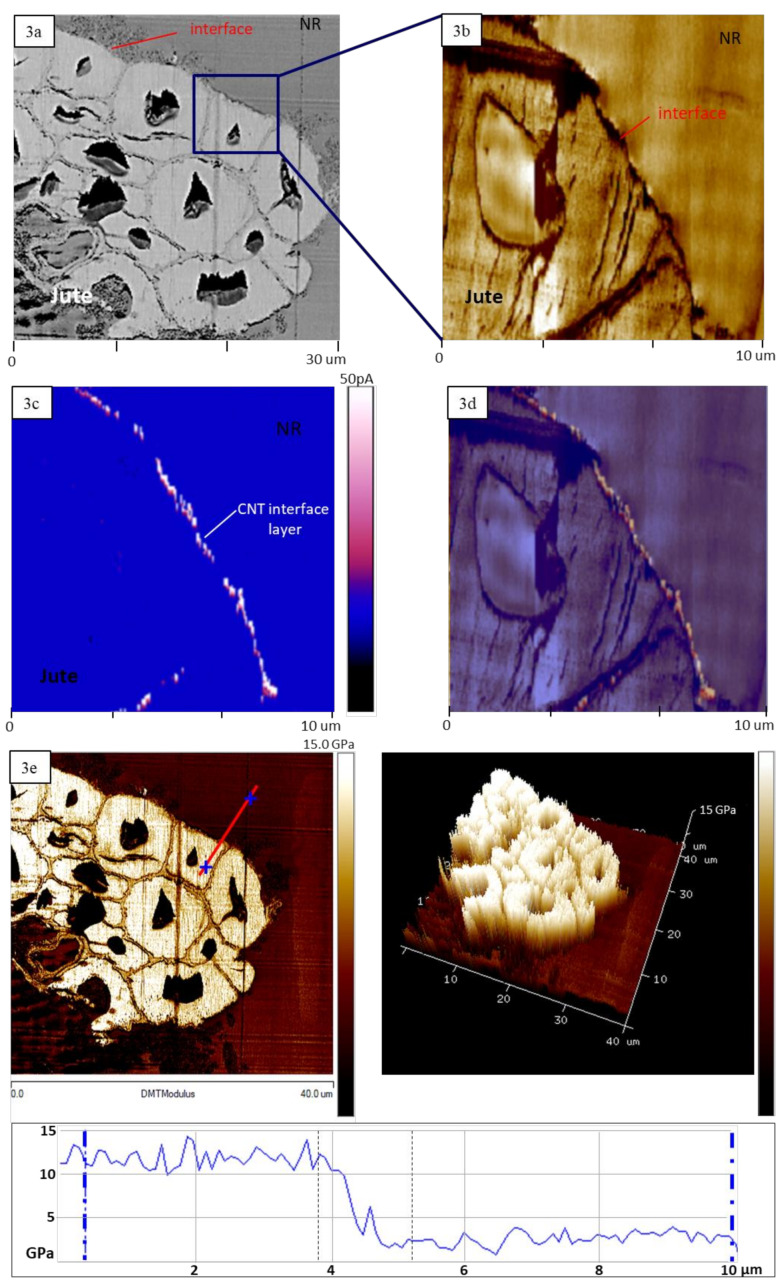
(**a**,**b**) Atomic force microscopy (AFM) phase images, (**c**) PeakForce TUNA current map of single JF-CNT/NR cross-section (scale: 50 pA, Vbias = 5 V) and (**d**) the overlay of Figure 3b,c images; (**e**) 2D and 3D peak force tapping quantitative nanomechanical (PFT-QNM) analysis of Derjaguin–Muller–Toporov (DMT) modulus map over the NR/JF-CNT cross-section and the corresponding section analysis (indicated red line).

Figure 3 shows the PeakForce TUNA atomic force microscopy images in conductive mode providing both the conductive map as well as the DMT modulus map over the region of investigation and specifically elaborating details about the CNT-rich interfacial region.

Figure 3a depicts an AFM phase image showing a general view of the NR/JF-CNT cross-section at a 30 × 30 μm^2^ area. Figure 3b represents also a phase image across the interphase (10 × 10 μm^2^ area), showing clearly the regions of fiber-interphase-NR matrix with phase contrast due to the different material’s properties. Figure 3c shows the c-AFM image demonstrating the nanoscale electrical current map. This image represents a qualitative sense of the electrical properties with a continuously conductive composite interphase due to the localization of MWCNTs at the interfacial region. Figure 3d is the overlay of Figure 3b,c images showing more precisely the path by which the electrical current passes through. This represents the mechanism that current flows also between the adjacent fibers in the NR matrix inducing the electrical conductivity observed for NR/JF-CNT bulk composites.

Figure 3e shows the PFT-QNM analysis and specifically the DMT modulus map of the NR/JF-CNT cross-section at a 40 × 40 μm^2^ area. A 2D and 3D image together with a section analysis are provided for better clarify and demonstration of the DMT modulus nanomechanical property mapping over the scanned area under investigation.

The AFM analysis fully corroborates the localization of CNTs at the composite interfacial region. This is in good agreement with the transmission electron microscopy interphase cross-section characteristics of a single NR/JF-CNT hierarchical composite that have been observed and presented in our previous study [15]. Specifically, the transmission electron microscopy cross section has shown densely packed CNTs at the interfacial region between the fiber and the rubber matrix with a thickness of 150–200 nm. This CNT layer which is a continuous interconnected assembly of CNTs around the fiber is responsible for the electrical characteristics observed by the c-AFM, as well as the bulk electrical conductivity endowed to the NR compounds.

### 3.3. Electrical Conductivity and Thermoelectric Properties of Bulk Natural Rubber (NR)/JF-CNT Hierarchical Composites

Carbon nanotubes are one-dimensional (1D) nanostructures with a tube-like morphology in which the electrons can propagate freely. The JF-CNT reinforcements turn the rubber into an electrical conductor after a critical concentration, well-known as the percolation threshold (Φ*c*), wherein a continuous network of filler is formed across the matrix and electron transfer can occur via a hoping and/or a tunneling mechanism [39]. The percolation threshold and conductivity depend strongly on the type of the polymer, type and aspect ratio of conductive filler, state of dispersion and distribution of fillers and degree of alignment [40]. The JF-CNT tested herein have a 6 wt.% CNT loading, as has been proven in our previous study by thermogravimetric analysis (TGA) experiments [15]. This makes after calculations a total amount of 0.6, 1.2 and 1.8 phr loadings of CNTs into the NR compounds for the 10, 20 and 30 phr NR/JF-CNT, respectively. Low *Φc* of single walled carbon nanotubes in polymer hosts has been reported from 0.005 vol.% to several vol.% [41]; however, such low *Φc* has not reported in any of the rubber matrices. Namely, Subramaniam et al. has reported ionic liquid-modified MWCNT nanocomposites of polychloroprene rubber with a *Φc* above 1 phr and a conductivity reaching ~10 S/m at 5 phr MWCNT loading [42].

**Figure 4:**
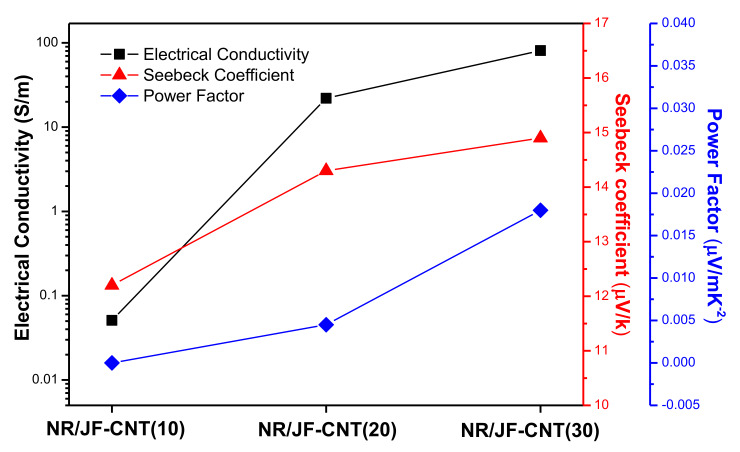
Electrical conductivity (*σ*), Seebeck coefficient (*S*) and power factor (PF) values of the NR/JF-CNT composites at 10, 20 and 30 phr JF-CNT loadings.

Figure 4 shows the electrical conductivity (*σ*) together with the Seebeck coefficient (*S*) and the derived power factor (*PF*) for the NR hierarchical composite filled with 10, 20 and 30 phr JF-CNT. The electrical conductivity of pristine rubber was found to be 6.5 × 10^−9^ S/m, while for the 10 phr loading the composite already is above the percolation threshold and becomes a conductor from its inherent insulating behavior, with a conductivity of 5.1 × 10^−2^ S/m. For the 20 phr and 30 phr loadings, the conductivity rises to 2.2 × 10^1^ S/m and 8.1 × 10^1^ S/m, respectively, being three orders of magnitude higher than the 10 phr composite. At 30 phr, the fibers are more densely packed in the elastomer matrix allowing better electron transport resulting in a higher electrical conductivity of the final hierarchical composite. The importance of this work and the beauty of short conductive hierarchical micro-/nano-structures as electrically conductive reinforcing fillers lies in the fact that at 30 phr filled NR (1.8 phr MWCNT content), the conductivity is 8 times higher than reported values of 5 phr filled rubber compounds [42] and is the highest conductivity in literature for rubber/CNT nanocomposites at such filler loadings.

The Seebeck coefficient for the three different composites is found to only slightly increase from the 10 phr composite (*S* = 12.2 μV/K) to 20 phr (*S* = 14.3 μV/K) and 30 phr (*S* = 14.9 μV/K), respectively. It is quite sensible that the Seebeck coefficient does not change for the different loadings, since the thermoelectric voltage produced by the bulk NR/JF-CNT material arises from the CNT-rich interphases that consist of MWCNT NC7000 type, while the Seebeck coefficient is known to be an inherent material’s property. However, it is interesting to mention that when the samples are getting far from their electrical percolation threshold i.e., at higher JF-CNT loading, the Seebeck slightly increases. A plausible explanation mechanism is that, upon electrical conductivity increase, the internal resistance of the sample decreases. Therefore, the thermoelectrically generated carriers (in our case “holes” since a positive Seebeck voltage has been measured) can find more conductive paths resulting further in a more efficient carrier transport from the “hot” to the “cold” side of the thermoelement exposed to the thermal difference (∆T). As such, a slightly higher voltage potential is measured yielding a slightly higher Seebeck coefficient, while this is in full agreement with our previous findings [43]. The highest power factor was found for the NR/JF-CNT(30) hierarchical composite (*PF* = 1.80 × 10^−2^ μW/m K^−2^).

## 4. Conclusions

The introduction of CNT-networks onto the surface of short JFs created a hierarchical micro-/nano-structured reinforcement, which induced electrical conductivity and thermoelectric properties to the manufactured 10, 20 and 30 phr filled NR compounds. The micron-scale natural filler is utillised as the “support” or the “template” to introduce the conductive and thermoelectric CNT nanoparticles into the NR composites, while for the 30 phr JF-CNT loading an extremely high conductivity of 81 S/m has been achieved compared to reported values for rubber/CNT nanocomposites at such loading levels. The highest power factor was found also for the NR/JF-CNT(30) hierarchical composite (*PF* = 1.80 × 10^−2^ μW/m K^−2^), mainly due to the conductivity enhancement at the 30 phr JF-CNT loading and not the Seebeck coefficient which remains almost the same for all NR/JF-CNT formulations. PF-QNM, c-AFM and TEM investigations demonstrated the nanostructured and biomimetic interphases created in the NR/JF-CNT composites, which are responsible for the electrical and thermoelectric properties endowed to the final composites. Finally, the JF-CNT showed a promising reinforcing effect as proven by the mechanical tensile tests with the optimal performance at the 20 phr loading.

The findings of this study can also trigger the development and design of hierarchical reinforcements of JFs or other micro-scale fibers (glass fibers, carbon fibers, etc.) with SWCNTs, graphene, etc. to create electrically conductive and thermoelectric interfaces; while avoiding issues of processing during mixing of nanoparticles in a polymer matrix.

The application of these novel elastomeric materials could be realized where good electrical and mechanical properties are required at lower nanofiller loadings, while these materials could be used as the next-generation stretchable thermoelectrics and potentially the building blocks to fabricate stretchable thermoelectric generator (TEG) devices.

## Figures and Tables

**Table 1:** The mixture formulation of natural rubber/jute fiber (NR/JF) composites.

Mix. No. and Composition *	1	2	3	4	5	6	7
NR (natural rubber)	100	100	100	100	100	100	100
ZnO (zinc oxide)	5	5	5	5	5	5	5
Stearic Acid	5	5	5	5	5	5	5
CBS (n-cyclohexyl -2- benzothiazole sulfenamide)	1.5	1.5	1.5	1.5	1.5	1.5	1.5
S (Sulfur)	1.5	1.5	1.5	1.5	1.5	1.5	1.5
JF-CNT (jute fibers coated with CNTs)	-	10	20	30			

* Numbers for components in recipe are in phr (parts per hundred rubber)

**Table 2:** Cure and physical properties of NR compounds.

Mix. No. and Cure Properties	S_min_ (dNm)	S_max_ (dNm)	t_2_ (min)	t_90_ (min)	Cure Rate Index *
NR	0.39	4.91	4.92	16.0	9.02
NR/JF-CNT_10	0.42	6.29	5.09	17.02	8.38
NR/JF-CNT_20	0.48	7.03	4.17	16.79	7.92
NR/JF-CNT_30	0.65	9.12	3.81	14.14	9.68

* The cure rate index (CRI) is given by the formula CRI = 100/(t_90_ − t_2_) and after calculations is given in the last column of Table 2.
